# Predictive value of platelet to lymphocyte ratio for in-hospital outcome of patients admitted with diagnosis of exacerbation of COPD with type II respiratory failure

**DOI:** 10.1097/MS9.0000000000003352

**Published:** 2025-05-12

**Authors:** Anand Kumar Deo, Kirti Kala Kharel, Deepshikha Kharel, Santa Kumar Das

**Affiliations:** a Department of Internal Medicine, Maharajgunj Medical Campus, Institute of Medicine, Tribhuvan University, Kathmandu, Nepal; b Nobel Medical College and Teaching Hospital, Biratnagar, Nepal; c Department of Pulmonology and Critical Care, Maharajgunj Medical Campus, Institute of Medicine, Tribhuvan University, Kathmandu, Nepal

**Keywords:** acute exacerbation, chronic obstructive pulmonary disease, in-hospital outcome, platelet to lymphocyte ratio, type II respiratory failure

## Abstract

**Introduction:**

Exacerbations of chronic obstructive pulmonary disease (COPD) are often associated with increased local and systemic inflammation. They tend to worsen the quality of life of sufferers and increase hospital admissions and mortality. Reliable and readily available biomarkers to identify the high-risk patients are necessary to facilitate early implementation of appropriate treatment strategies.

**Methods:**

This prospective cohort study, conducted on 110 patients, aimed to study the predictive value of platelet to lymphocyte ratio (P/L) at the time of admission for in-hospital outcome of patients admitted with a diagnosis of exacerbation of COPD with Type II respiratory failure. Patients were followed up during their entire stay at the hospital and their blood tests, clinical course and outcome were recorded. The data obtained were recorded in an Excel sheet and analyzed using Statistical Package for Social Science (SPSS) version 25. The work has been reported in line with the STROCSS criteria.

**Results:**

Among 110 patients, 11 (10%) died during hospitalization. Mean P/L at the time of admission was 214.56 ± 84.13 among the patients who eventually died at hospital and 152.53 ± 121.19 among those who were discharged (*P* = 0.006).

**Conclusion:**

Our study shows that the mean P/L at the time of admission was significantly higher in COPD patients with type II respiratory failure who died during hospitalization compared to those who were discharged. These findings suggest that the P/L could serve as potential prognostic biomarker for in-hospital mortality risk in COPD exacerbations.

## Introduction

Chronic obstructive pulmonary disease (COPD) is chronic inflammatory state. The state of inflammation is even higher during acute exacerbations^[^1^]^. Pulmonary inflammation results in the entry of cytokines including tumor necrosis factor-alpha (TNF-α), interleukin (IL)-1, IL-6, and IL-8 into the systemic circulation, leading to increased acute phase proteins as CRP, fibrinogen, serum amyloid A, and surfactant protein D^[^2^]^. Blood leukocyte count is another marker of inflammation. As a compensatory mechanism for the control of infective presentation, certain cytokines such as IL-8 released by macrophages, which are the first-line defense mechanism against infections, stimulate hematopoiesis, thereby increasing blood leukocyte counts^[^3^]^. Platelets also have role in modulation of inflammatory response. Although there are only a limited number of studies on platelet counts in COPD patients, thrombocytopenia has been reported in COPD patients with acute exacerbation and has been associated with poor prognosis and increased mortality^[^4^]^.

**HIGHLIGHTS**

- Exacerbations of chronic obstructive pulmonary disease (COPD) are common and has high mortality rate.
- It is paramount to identify patients of COPD exacerbation with high risk of deterioration and mortality.
- Platelet to lymphocyte ratio is a readily available marker of inflammation.

Ongoing inflammation has detrimental effects on the outcome of patients admitted with exacerbation of COPD. It is paramount to identify patients with higher risk of mortality to facilitate early implementation of appropriate treatment strategies. Biomarkers have potential value as prognostic indicators in COPD, though their utility has been limited by disease heterogeneity and the influence of comorbidities^[^5^]^. While C-reactive protein, Procalcitonin are established markers of inflammation they are not routinely available and expensive as well. In the quest for less expensive and routinely available biomarkers, an increasing body of evidence suggests that neutrophil to lymphocyte ratio (N/L) and the platelet to lymphocyte ratio (P/L) can significantly predict adverse outcomes in patients with exacerbation of COPD. Also, current evidence supporting the presence of significant associations with several pre-defined adverse clinical outcomes in patients with exacerbation of COPD is stronger for the N/L than P/L. However, such studies are limited in number and even less for P/L. Furthermore, only a handful of studies have discussed the cutoff values for P/L to predict poor outcome of such patients

This study aims to examine the association between the platelet to lymphocyte ratio and the in-hospital outcome of patients with exacerbation of COPD and type II respiratory failure. It hopes to add a new tool to the armamentarium of physicians for predicting outcomes and enabling them to use multiple, inexpensive and readily available predictive models in unison to decide on treatment strategies for patients admitted with COPD exacerbation and type II respiratory failure. Additionally, this study aims to determine the cutoff value for P/L to predict in-hospital mortality using the ROC curve. This study will also add to the limited existing literature on prognostic utility of P/L for patients of COPD exacerbation.

## Research design and methodology

This prospective cohort study was conducted in the Department of Pulmonology and Critical Care of a top-tier tertiary care center of the country over 10 months from August 2022 to May 2023. The work has been reported in line with the STROCSS criteria^[^6^]^. It has been registered in Research registry with UIN research registry 10586.

### Sample size calculation

In the preceding 10 months, total of 304 patients were admitted in the same hospital with the diagnosis of exacerbation of COPD with type II respiratory failure. To study the predictive value of P/L, we’ve considered in-hospital mortality as primary outcome. In the study conducted by Bam *et al* on “Factors determining Outcomes in Hospitalized Patients with Acute Exacerbation of Chronic Obstructive Pulmonary Disease” at same hospital, in-hospital mortality was found to be 12.7%^[^7^]^. Therefore, using Daniel formula

Sample size (*n*) = *Nz*^2^*p*(1 − *p*)/*d*^2^(*N* − 1) + *z*^2^*p*(1 − *p*)

where *n* = sample size with finite population correction, *N* = finite population size (304), *Z* = *z* statistic for a level of confidence (1.96), *P* = expected proportion (12.7), *D* = precision (0.05), and therefore the sample size (*n*) = 110.

Sample population was selected by convenient sampling method over 10 months. 110 consenting adult patients meeting the inclusion and exclusion criteria were included in the study.

The consensus definitions used for the purpose of study has been mentioned in Supplementary Appendix (available at: http://links.lww.com/MS9/A827).

### Inclusion and exclusion criteria

- Inclusion criteria:
  - Age > 40 years.
  - Admitting diagnosis of exacerbation of COPD with type II respiratory failure. A patient will be said to have COPD for the purpose of this study if treating physician has made a diagnosis of COPD based on clinical scenario of persistent, progressive dyspnea that is characteristically worse with exertion, chronic cough, or sputum production, a history of recurrent LRTIs and/or a history of exposure to risk factors for the disease, with characteristic findings on examination and typical radiographic findings, with/without spirometry confirmation of the disease.
  - Consent for enrollment in study.
- Exclusion criteria:
  - Patients with restrictive lung disease. The treating physician decision on nature of respiratory illness will be taken into account as final diagnosis.
  - Patients with non-COPD cause for type II respiratory failure.
  - Acute respiratory distress syndrome.
  - Massive pulmonary embolism.
  - Malignancy.

### Consent and ethical considerations

Patients received standard of care as per treating physician’s discretion. This study did not add any economic burden to the patient. A paragraph emphasizing the confidentiality of patient data and the option to withdraw from the study at any time was provided to the person consenting for the study. They were also informed of their right to withdraw from the study, without incurring any negative consequences. Written informed consent was obtained from the study participants or assigned relatives prior to the study’s commencement.

## Statistical analysis

The data were entered in a Microsoft excel sheet and converted into IBM SPSS version 25 for statistical analysis. The categorical data were represented in the form of frequency and percentage. Results on continuous data were represented as mean ± standard deviation. Significance of association between categorical variable with dichotomous values and continuous variable was tested with Mann–Whitney U test. Results were interpreted at 95% level of confidence and *P* <0.05 was considered as significant.

## Results

A total of 118 patients with admitting diagnosis of exacerbation of COPD with type II respiratory failure were enrolled. However, 8 of them were later diagnosed as case of Bronchiectasis by treating physician and were excluded from analysis. Total of 110 patients were included in the analysis.

### Baseline characteristics of the study population

The mean age of patients was 69.93 ± 8.92 years, majority (45.5%) belonging to age group 70–80 years. 66.4% of the population were female. 34.5% of sample population were already on domiciliary oxygen prior to presentation. 35.5% of enrolled patients had history of frequent exacerbations. 36.4% of sample population had baseline dyspnea of mMRC grade 3 and 60.0% of population had baseline dyspnea mMRC grade 2. 67.3% of sample population were former smoker and 23.6% were current smoker. The data are summarized in Table 1.

**Table 1:** Comparison of baseline characteristics between people with various smoking status

		Never smoker n = 10	Former smoker n = 74	Current smoker n = 26
Mean age (years)		73.4 ± 6.0	70.6 ± 8.9	66.7 ± 9.3
Frequency of exacerbations	Infrequent exacerbator	7 (70%)	50 (67.6%)	14 (53.8%)
	Frequent exacerbator	3 (30%)	24 (32.4%)	12 (46.2%)
Baseline mMRC	1	0 (0%)	4 (5.4%)	0 (0%)
	2	4 (40%)	44 (59.5%)	18 (69.2%)
	3	6 (60%)	26 (35.1%)	8 (30.8%)
Mode of O2 delivery prior to presentation	None	9 (90%)	46 (62.2%)	17 (65.4%)
	Domiciliary O2	1 (10%)	28 (37.8%)	9 (34.6%)

### Outcome and analysis

Eleven patients (10%) of study population succumbed to death at hospital. Twenty-seven (24.5%) patients required new intensive respiratory support (ICU transfer and subsequent invasive/non-invasive ventilation) during hospital stay. Nine (8.2%) patients required mechanical ventilation. While 25.9% of those requiring intensive respiratory support died in hospital, only 4.8% of those not requiring intensive respiratory support died in hospital. 33.3% of those requiring mechanical ventilation (MV) and 7.9% of those not requiring MV died in hospital. Median duration of stay at hospital was 7 days.

Platelet to lymphocyte ratio (P/L mean 214.56 ± 84.13 vs. 152.53 ± 121.19, *P* = 0.006) at the time of admission was significantly higher in those who eventually died at hospital compared to those who were discharged. Mean P/L was numerically higher among those who did not require intensive respiratory support or mechanical ventilation compared to their respective counterparts but the values could not attain statistical significance. The data are summarized in Table 2.

**Table 2:** Comparison of Platelet to lymphocyte ratio (P/L) at the time of admission of patients admitted with diagnosis of exacerbation of COPD with type II respiratory failure

		No. of patients	P/L (mean ± SD)	*P* value
In-hospital Outcome	Died	11	214.56 ± 84.13	**0.006**
	Discharged	99	152.53 ± 121.19	
Requirement of Intensive respiratory support	Required	27	122.39 ± 67.25	0.051
	Did not require	83	170.56 ± 129.88	
Requirement of Mechanical ventilation	Required	9	119.24 ± 65.82	0.232
	Did not require	101	162.26 ± 122.4	

Mann–Whitney U test applied to test statistical significance.

**Figure 1.**
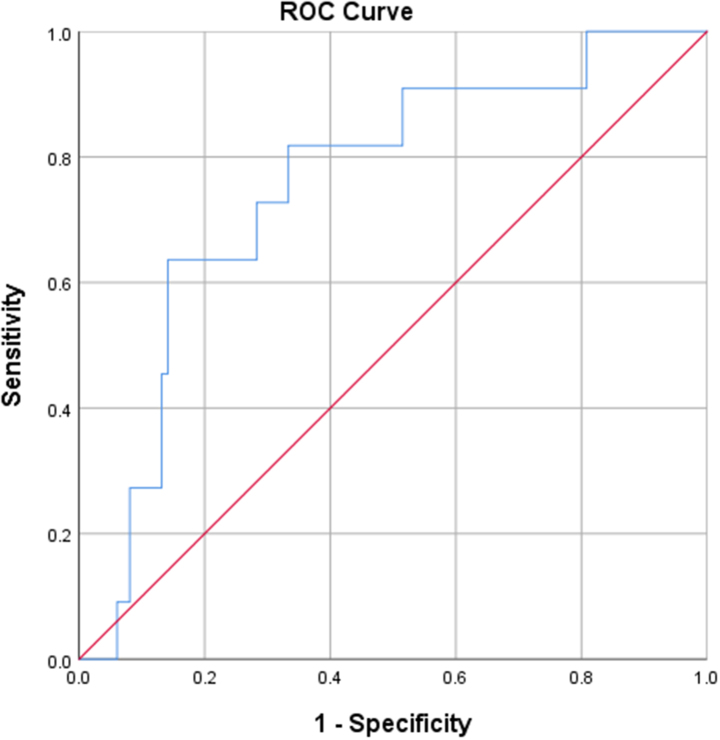
Receiver operating characteristic curve (ROC) of P/L and in-hospital mortality in patients admitted with exacerbation of COPD and type II respiratory failure.

Prognostic utility of P/L was assessed by calculating its sensitivity and specificity using the ROC curve. The area under the ROC curve for in-hospital death and P/L was 0.754 (95% CI, 0.612–0.896, *P* = 0.006) as shown in Fig. 1. ROC analysis also identified platelet to lymphocyte ratio (P/L) ≥ 208.64 had a sensitivity of 63.6% and specificity of 85.9% in predicting in-hospital mortality.

## Discussion

Exacerbation is leading cause of hospitalization and mortality in COPD patients. While COPD is a chronic inflammatory state, inflammation is even more during exacerbations. CRP and procalcitonin are established biomarkers, but they have limited significance in exacerbations of viral etiology. They’re expensive and not routinely available as well. The quest for easily obtainable, readily available and affordable biomarker for prognostication of COPD exacerbation patients is ongoing. This study aimed to study the predictive value of Platelet to lymphocyte ratio (P/L) for in-hospital mortality of patients’ admitted with exacerbation of COPD and type II respiratory failure.

The immune system relies heavily on lymphocytes, and lymphocytopenia has been linked to increased infection and mortality rates in sepsis^[^8^]^. Lymphocytopenia has been associated with all-cause mortality in COPD patients as well^[^9,10^]^. Apoptosis of lymphocytes is also common in COVID 19 and various other viral infections that are common aggravating factors for COPD^[^11^]^. Peripheral blood lymphocytes may be decreased in the elderly^[^12^]^ and older age is also a significant risk factor for COPD mortality^[^13^]^. In a retrospective study of 384 patients with COPD exacerbation, non-survivors were found to have lower lymphocyte counts compared to those who were discharged (0.84 ± 0.89 × 10^9^/L vs. 1.09 ± 0.60 × 10^9^/L, *P* = 0.020)^[^14^]^. Platelets also play a key role in the modulation of inflammation and immune responses. Platelet p-selectin expression and the subsequent formation of platelet-leukocyte aggregate up regulate leukocyte pro-inflammatory functions. In addition, platelet α-granules contain several types of cytokines with predominant pro-inflammatory effects^[^15,16^]^. Various studies have shown higher P/L in COPD patients during exacerbation compared to after treatment of exacerbation implying P/L is indeed a reliable marker of inflammation and exacerbation in COPD patients. In a study by Fayiad and Amer, P/L was found to be 223.59 ± 97.14 during COPD exacerbation and 105.63 ± 18.77 after a month of discharge. P/L was also found to have positive correlation with CRP, an established biomarker of inflammation, in patients with COPD exacerbation (r = 0.320, *P* = 0.004)^[^17^]^. However, in this study, the patients were not classified based on etiology of exacerbation. CRP is markedly elevated in bacterial infection and not so much in viral infections. Viral infections make up the majority of exacerbation and therefore only a weak correlation was obtained between P/L and CRP in aforementioned study.

In our study, mean Platelet to lymphocyte ratio (P/L) and at admission was significantly higher in the group that died in hospital (214.56 ± 84.13 vs. 152.53 ± 121.19, *P* = 0.006). A similar finding of statistically significant high mean P/L (362.4 vs. 240.8, *P* = 0.033) and (272.26 ± 184.28 vs. 198.1 ± 140.8, *P* = 0.004) was found among non-survivor group in two different studies conducted in China^[^14,18^]^. In our study, P/L ≥208.64 had a sensitivity of 63.6% and specificity of 85.9% in predicting in-hospital mortality. A retrospective study with 303 patients of COPD exacerbation found that, at a cut-off value of 182.68, the sensitivity and specificity of the P/L in predicting in-hospital mortality were 64.86% and 58.27%, respectively, with an AUC of 0.64 implying a lower predictive accuracy compared to our study^[^18^]^. Similarly, in a retrospective cohort study of 181 patients admitted with COPD exacerbation, ROC analysis identified platelet to lymphocyte ratio (P/L) ≥235 had a sensitivity of 63% and specificity of 74% in predicting 90-day mortality^[^19^]^. In our study, mean P/L was numerically higher among those who did not require intensive respiratory support or mechanical ventilation compared to their respective counterparts but the values could not attain statistical significance. This is indirectly supported by another study where a statistically significant higher P/L was observed in patients with COPD exacerbation requiring mechanical ventilation compared to those not requiring mechanical ventilation (mean P/L 281.04 ± 101.17 vs. 194.32 ± 81.42, *P* < 0.001)^[^17^]^. While our study indicates that the P/L is a simple and useful biomarker for predicting in-hospital mortality in patients with COPD exacerbation and type II respiratory failure, bearing in mind the current limited evidence, we suggest that results of the P/L alone should be explained with caution and best used in conjunction with traditional inflammatory maker such as CRP to ensure better prognostic accuracy.

## Conclusion

In our study of patients admitted with diagnosis of exacerbation of COPD with type II respiratory failure, the in-hospital mortality was 10%. Patients who died in hospital had significantly higher platelet to lymphocyte ratio compared to those who were discharged. Identifying the high-risk characteristics and parameters among patients to assess risk of mortality is of utmost importance to reduce the poor outcome of exacerbation. P/L at the time of admission was found to be easily obtainable and reliable harbingers of worse outcome in COPD patients admitted with exacerbation and type II respiratory failure. Studies with larger cohort and with established biomarkers as comparators are essential to further validate the findings of this study.

## Data Availability

The study data has not been shared.

## Limitations

1. The small sample size limits the power of the study.
2. A physician labeled diagnosis of COPD without spirometry is likely to not include all cases of COPD and at the same time likely to include some cases with non-COPD exacerbations as well.
3. Infective and non-infective triggers of exacerbation were not separated and therefore marker of inflammation like P/L cannot be ascertained to chronic inflammatory nature of COPD exclusively.
4. Correlation between P/L and established markers of inflammation like CRP or stronger counterpart N/L was not studied.

## Recommendations

1. Further studies with larger sample size, including cohort that had been previously diagnosed as COPD based on clinical scenario along with spirometry and longer duration of follow up are necessary.
2. Prospective cohort study including COPD patients without exacerbation and with exacerbation as well as stratification based on cause of exacerbation (infective/non infective) are necessary to analyze the predictive value P/L.
3. Further studies comparing P/L with established markers of inflammation like CRP or stronger counterpart N/L needs to be studied.

## Research registration unique identifying number (UIN)

researchregistry10586.
